# FDA-Regulated Clinical Trials vs. Real-World Data: How to Bridge the Gap in Pain Research

**DOI:** 10.3390/brainsci15101119

**Published:** 2025-10-18

**Authors:** Anthony Reyes, Mohummed Malik, Malik Sahouri, Nebojsa Nick Knezevic

**Affiliations:** 1 Department of Physical Medicine and Rehabilitation, Rush University Medical Center, Chicago, IL 60612, USA; anthony_j_reyes@rush.edu; 2 Department of Anesthesiology, Advocate Illinois Masonic Medical Center, Chicago, IL 60657, USA; mohummed.malik@aah.org (M.M.); malik.sahouri@aah.org (M.S.); 3 Department of Anesthesiology, University of Illinois, Chicago, IL 60612, USA; 4 Department of Surgery, University of Illinois, Chicago, IL 60612, USA

**Keywords:** randomized controlled trials, FDA clinical trials, real-world data, real-world evidence, efficacy-effectiveness gap, chronic pain research

## Abstract

Randomized controlled trials (RCTs) have been regarded as the gold standard for evaluating the efficacy of treatments for chronic pain and are the foundation for regulatory approval and guideline development. However, their restrictive design and dependence on idealized populations can limit their applicability to the diverse patients seen in routine chronic pain management. Real-world data (RWD), collected from electronic medical records, registries, claims databases, and digital health platforms, can offer a more comprehensive view of treatment adherence and safety that RCTs often overlook. A key issue in pain medicine is the efficacy–effectiveness gap, where discrepancies exist between the outcomes of therapies and interventions in RCTs versus in real-world practice due to variations in patient populations and adherence. Bridging this gap ensures that observed improvements align with patients’ preferred outcomes and functional goals. Integrating the strengths of RCTs and RWD provides a more comprehensive evidence base to guide clinical decision-making, influence reimbursement policies, and develop equitable guidelines. The primary aim of this paper is to identify factors used in FDA-regulated RCTs and RWD that could be implemented or enhanced in everyday practice to deliver more holistic and patient-centered care in the management of chronic pain.

## 1. Introduction

Randomized controlled trials (RCTs) have been regarded as the gold standard for assessing the efficacy of pharmacologic and interventional treatments in medicine. Through randomization and strict inclusion criteria, RCTs minimize bias and maximize internal validity, which in turn influences regulatory approval and clinical guidelines [1,2,3]. RCTs have played a key role in shaping the current therapeutic landscape in pain medicine; however, their restrictive design can limit generalizability and raise questions about the practical effectiveness of translating RCT findings into everyday chronic pain management [3,4,5,6].

In contrast, real-world data (RWD), sourced from electronic medical records (EMRs), patient registries, insurance claims, and digital health platforms, offers observations of patient outcomes beyond the controlled setting of RCTs [3,7,8,9]. When analyzed to produce real-world evidence (RWE), these data represent diverse populations and complex comorbidities that are more typical of patients seen in everyday clinical practice [10,11]. While RCTs are studies designed to determine the efficacy of a drug or intervention under ideal conditions, RWD and RWE evaluate the effectiveness of these interventions and their impact in routine clinical practice while accounting for factors such as patient adherence and comorbidities [10]. 

A major challenge in chronic pain medicine is the efficacy–effectiveness gap, which is the disconnect between the significant results seen in RCTs and the inconsistent outcomes reported in clinical practice [1,11]. Pain patients are a heterogeneous group with multiple medical and psychosocial comorbidities, along with variable responses to treatment [4]. However, strict RCT inclusion criteria often exclude diverse patients who are more representative of those found in real-world settings [3,6]. Therefore, the benefits of pain treatments demonstrated in trials can be less defined when applied to broader patient populations. 

The primary aim of this paper is to identify factors used in U.S. Food and Drug Administration (FDA)-regulated RCTs and RWD that could be implemented or enhanced in everyday practice for better patient-centered pain management. Bridging the gap between FDA-regulated clinical trials and RWD is essential for advancing pain research. By integrating the internal validity of RCTs with the external validity of RWD, researchers can gain a more comprehensive understanding of treatment effectiveness, safety, and long-term outcomes [7,8]. Utilizing this approach can improve clinical guidelines and influence holistic treatment strategies, supporting evidence-based decisions that better reflect the realities of managing chronic pain [1,12].

## 2. RCTs vs. RWD

### 2.1. Randomized Control Trials

Precise methodology, including random allocation, strict eligibility criteria, and standardized intervention delivery, gives RCTs their high internal validity and establishes their role as the foundation of evidence-based guidelines [1,3]. Randomization minimizes confounding and ensures that differences in outcomes can be attributed to the intervention being studied. Blinding reduces the risk of measurement bias, which is essential in pain research where subjective reporting is central to outcome assessment [13]. Additionally, standardized interventions and prespecified protocols allow for reproducibility across sites, which further reinforces confidence in the validity of findings [14]. These features allow RCTs to provide the most reliable estimates of causal treatment effects. However, this rigor also introduces rigidity, which limits their general applicability. Due to strict eligibility criteria designed to minimize confounding, participants are typically selected from narrowly defined populations, excluding individuals with comorbidities, psychiatric conditions, polypharmacy, or socioeconomic complexity, all of which represent a large proportion of those treated for chronic pain in clinical practice [6,15]. As a result, study populations are more homogeneous than the patients clinicians actually encounter, creating a gap between trial results and everyday clinical realities. For example, an intervention proven efficacious in a carefully selected cohort may be less effective, or even less safe, when applied to individuals with multimorbidity or psychosocial instability [3].

RCTs monitor participants under idealized conditions that can overestimate effectiveness. The intensive oversight and reinforcement of therapy compliance seen in studies rarely reflect everyday clinical practice [1]. In pain management, adherence varies, follow-up is inconsistent, and physicians attempt to tailor decisions to each patient’s needs. To address this variability, providers use their best clinical judgement, which in turn needs to be based on reliable data. For example, the most potent drug is not necessarily an ideal option in a patient with specific comorbidities or psychosocial issues; therefore, treatment algorithms need to be adjusted for that individual. These deviations from trial conditions can impact both safety and effectiveness profiles, sometimes in unexpected ways that were not predicted by trial evidence. Kim et al. (2018) acknowledged that while RCTs are considered the most reliable form of evidence, they “may fail to reflect the actual clinical site sufficiently” and risk bias when applied outside their constrained sample frameworks [3].

Finally, RCTs are limited by their scope and duration. Chronic pain treatments often require years of ongoing management, with outcomes influenced not only by immediate analgesic effects but also by long-term functional quality of life and evolving safety considerations [13,16,17]. Most RCTs are not designed to capture these longitudinal trajectories. Sample sizes are usually too small to detect rare or delayed adverse events, and follow-up periods are often too short to assess the durability of benefits [7]. These limitations do not diminish the value of RCTs, but instead highlight the importance of interpreting their findings with caution and supplementing them with other types of evidence [18].

### 2.2. Real-World Data

RWD has become an essential supplement to RCTs, driven by advancements in EMRs, patient registries, and patient-generated data from wearable devices and mobile health platforms [3,7,8]. Unlike trials, RWD captures the complexity of real-world clinical practice, allowing researchers and providers to observe treatment as it naturally occurs, offering a more authentic view of how pain therapies are delivered and experienced outside highly controlled trial settings [12]. One key advantage of RWD is its ability to reflect the nuances of everyday practice. Unlike RCTs, which exclude patients who do not meet strict criteria, RWD includes individuals who reflect the true diversity of the chronic pain population [15]. This inclusivity allows for the evaluation of treatment outcomes in groups that are typically underrepresented in trials. As Bartlett et al. (2019) highlight, RWD reflects real-world clinical scenarios, such as missed appointments, therapy non-compliance, and clinician-driven modifications, which can affect the long-term effectiveness of treatments but are often overlooked in traditional trials [1].

EMRs provide longitudinal data on pain scores, functional outcomes, comorbidities, and medication use. Meanwhile, large-scale registries and claims databases have expanded access to population-level data through the systematic collection of patient-reported outcomes (PROs) and evaluation of the effectiveness of various treatments across populations [5,10,11]. The value of these data expands beyond measuring average treatment effects. RWD can identify subgroups of patients who respond differently to the same therapy, allowing for a more personalized approach to pain management. It can also reveal safety concerns that only emerge after widespread adoption of a drug or device [15]. In these ways, RWD addresses critical gaps left by RCTs, offering further knowledge of long-term effectiveness and applicability that can strengthen both clinical and regulatory decision-making.

Despite these advantages, RWD presents its own methodological challenges. The lack of randomization makes observational studies susceptible to selection bias and systematic differences between patients who receive a therapy and those who do not [19,20]. These differences not only reflect measurable demographics or comorbidities, but also subtle clinical judgments and patient qualities that impact treatment decisions in ways not reflected in datasets. As a result, comparisons based on RWD can risk attributing effects to the treatment itself when they may actually be due to underlying differences in patient characteristics. This issue is critical in pain medicine, where prescribing decisions are influenced by factors such as symptom severity, psychosocial effects, or prior treatment failures [15,20]. To minimize confounding and selection bias in real-world studies, techniques such as propensity score matching (PSM) are considered. PSM balances characteristics between patients entering various treatment arms and observes the outcomes in these similar patients [21]. However, propensity score matching itself limits the generalizability of a real-world study, as data needs to be grouped and therefore narrows the patient population [21].

Data quality represents another significant limitation. Clinical information recorded in EMRs is collected primarily for documentation and billing, not research. Therefore, pain scores and functional measures may be recorded inconsistently, and adverse events can go undocumented if they are not the primary focus during a clinical encounter [18]. Although claims data can be valuable for identifying healthcare utilization patterns, they offer limited clinical relevance and cannot reliably reflect key outcomes in pain research, such as quality of life or psychosocial functioning [18]. Additionally, the heterogeneity of real-world practice that gives RWD its strength can also complicate interpretation. Variations in clinician expertise, patient compliance, and treatment sequencing lead to results that may not be generalizable beyond the specific healthcare systems or populations studied [11,18]. Privacy regulations and fragmented data systems further limit efforts to link datasets across different settings, restricting the ability to create comprehensive longitudinal records [5,15].

RCTs and RWD each offer complementary strengths (Table 1). RCTs excel at establishing causal efficacy under tightly controlled conditions, while RWD provides a broader view of effectiveness, safety, and utilization in everyday practice. RWD can reinforce, expand, or even replicate trial findings; however, it is essential to carefully choose factors to evaluate to achieve results that can be translated clinically. RWD can guide RCTs by predicting clinical outcomes and aiding in studies that RCTs cannot ethically or practically conduct [3]. Some questions, such as long-term safety in broad populations or outcomes in high-risk groups, are not suitable for RCTs but can be explored through real-world investigations [1]. For example, RCTs often struggle to demonstrate efficacy in the treatment of neuropathic pain; however, RWD could offer evidence to update product labels or treatment recommendations when randomized data are limited or inconclusive [5]. Combining randomized and observational approaches offers the strongest path forward: rigorously designed RCTs to establish causal relationships, complemented and extended by RWD to assess real-world relevance and effectiveness in routine care [1,3].

## 3. Efficacy–Effectiveness Gap

The efficacy–effectiveness gap refers to the difference between outcomes seen in RCTs and those achieved in everyday clinical practice. Understanding this gap is particularly essential in pain medicine due to the complex, multifactorial nature of chronic pain conditions, variability in how patients respond, and the influence of psychosocial factors that are often underrepresented in clinical trials [10,11,15,18,22]. Unlike most therapeutic RCTs and RWD, which utilize biomarkers or physiological endpoints as primary outcomes, chronic pain studies lack universally accepted objective measures. Pain trials rely on subjective individual experiences to define clinically significant changes that will ultimately be generalized to the greater population [22]. Due to the nature of high internal validity and limited generalizability found with RCTs, interventions demonstrating strong efficacy in trials may underperform when applied to broader, more heterogeneous patient populations in real-world settings [15]. This gap has important implications for clinical decision-making, as relying solely on trial data can overestimate treatment effectiveness by neglecting real-world patient factors. Understanding this distinction is necessary to avoid overinterpreting efficacy and to develop treatment strategies that are feasible and effective across various patient groups.

Several factors contribute to the efficacy–effectiveness gap in pain research, some of which are listed in Table 2. Patient heterogeneity is a major factor, as real-world populations include groups that tend to be excluded from RCTs, such as older adults, individuals with multiple comorbidities, or those taking concomitant medications [11,18,23]. Variability in treatment adherence, differences in dosing strategies, provider or patient preferences, and inconsistent access to multidisciplinary care further widen this gap. The subjective and multidimensional nature of pain complicates measurement as standardized pain scales used in trials may not fully capture fluctuations in intensity, functional impairment, or quality-of-life outcomes experienced by patients outside the trial setting [23]. Socioeconomic factors, cultural differences, and patient expectations also affect both reporting and treatment response, creating additional layers of variability. Structural and systemic factors, including limited follow-up, differences in clinician expertise, and resource constraints, further influence the translation of trial findings into routine practice [11,18]. Overall, these factors demonstrate the challenges of implementing tightly controlled trial results into real-world pain management for diverse patient populations.

## 4. How Is Pain Measured?

Accurate and reliable assessment of pain is essential for both FDA-regulated clinical trials and RCTs. Because pain is inherently subjective and multidimensional, the choice of measurement instruments strongly influences trial outcomes, regulatory approval, and ultimately the translation of findings into clinical practice. The FDA has historically emphasized the use of validated, patient-reported outcome measures, but current research highlights the importance of balancing simplicity and clinical interpretability in tool selection [24,25]. Knowledge of how pain is measured in clinical trials can provide a more comprehensive understanding of the discrepancies that arise when translating treatment efficacy to effectiveness in the context of routine clinical pain management.

Unidimensional pain intensity scales are the most-used primary outcome measures, specifically the Visual Analog Scale (VAS) and Numeric Rating Scale (NRS). These tools are favored for their ease and sensitivity to change in both acute and chronic pain contexts. The VAS is a 100 mm line labeled with “no pain” at one end and “worst imaginable pain” at the other, providing continuous data that are highly sensitive to subtle shifts in patients’ perception of pain. The NRS, scored from 0 to 10, is easier to administer and has demonstrated strong validity and responsiveness across different patient populations [26]. Furthermore, unlike the VAS, the NRS does not require the use of paper or an electronic device. Both scales are well-suited to meet FDA requirements for primary efficacy outcomes and remain the standards in the development of pain treatments [25].

A recent study emphasized the importance of examining percentage reductions in pain, rather than relying solely on raw intensity changes. Fink et al. (2023) evaluated and analyzed two related but distinct measures: patient-reported percentage pain reduction (PRPPR) and calculated percentage pain reduction (CPPR) [27]. Although both metrics provide meaningful information, PRPPR better reflects patient perception of improvement, while CPPR provides a standardized calculation based on baseline and endpoint scores [27]. These tools together offer complementary insights and may serve as more detailed efficacy endpoints in FDA trials.

In addition to pain intensity, multidimensional instruments provide a better understanding of the broader impact of pain on patients’ lives. The McGill Pain Questionnaire (MPQ) and the Brief Pain Inventory (BPI) are two of the most common tools in this category. The MPQ assesses the sensory and emotional aspects of pain through descriptors that reflect patients’ qualitative experiences. Meanwhile, the BPI measures both pain severity and the degree to which pain disrupts daily functioning [25]. These tools are useful in chronic pain studies, as the complexity of the condition extends beyond numeric intensity scores. By integrating multidimensional questionnaires, trial designs can analyze treatment effects from a more comprehensive viewpoint and develop patient-centered approaches that are increasingly recognized by the FDA and international regulatory bodies [25].

Functional disability and quality-of-life outcomes represent another critical domain in clinical trial measurement. Instruments such as the Oswestry Disability Index (ODI) and the Roland–Morris Disability Questionnaire (RMDQ) are among the most validated and widely accepted measures of functional impairment, especially in populations with musculoskeletal and low back pain [24]. These tools evaluate limitations in mobility, self-care, and daily activities, which are domains directly relevant to both clinical practice and patient well-being. Not only do they help ensure that trials measure reductions in pain intensity, but they also address the functional and psychosocial impacts of chronic pain [24].

Despite the widespread use of the mentioned self-reported outcome measures, several important methodological issues are important to consider in tool selection. Younger et al. (2009) highlight the ongoing tension between statistical and clinical significance, emphasizing that outcomes should not only show detectable changes on a scale but also reflect improvements that are clinically meaningful to patients [25]. This has led to increased focus on the concept of minimal clinically important differences (MCIDs) when analyzing trial data [25,28]. MCIDs act as thresholds that differentiate statistical changes from improvements that patients perceive as valuable in their daily lives, such as improvement in pain, returning to work, achieving restful sleep, or reduction in medication use [28]. Establishing and validating these thresholds in pain research helps ensure treatment effects are interpreted in a patient-centric manner, ultimately aligning trial outcomes with real-world clinical benefits. However, a limitation of utilizing MCIDs in real-world studies is analyzing these differences in relation to pain scores. For example, the MCID for NRS is highly dependent on patients’ baseline pain scores and psychosocial factors within the patient population, which makes it challenging to standardize what would constitute a meaningful change across clinical settings [28].

Additionally, the logistics of data collection require careful consideration. Instruments that are overly lengthy or burdensome may compromise compliance and reduce data quality. Conversely, shorter, validated measures, when administered through electronic or home-based platforms, can enhance ecological validity and patient engagement [25].

## 5. Responder Thresholds

Pain medicine researchers and regulators, including the FDA, as well as insurance companies emphasize the importance of clinically meaningful thresholds or benchmarks that differentiate numerical changes from improvements that patients perceive as beneficial in daily life [14,29,30]. These thresholds guide trial design, outcome interpretation, and regulatory approval for treatments, ensuring that observed effects are not only measurable but also relevant to patients. By defining what constitutes as “meaningful benefit”, these thresholds create a shared language for patients, clinicians, and regulators to assess the value of new pain treatments.

The Initiative on Methods, Measurement, and Pain Assessment in Clinical Trials (IMMPACT) issued recommendations in 2005 to establish core outcome measures and define thresholds for clinical significance in pain trials [14]. IMMPACT determined that a ≥30% reduction in pain intensity indicates a “moderately important improvement,” while a ≥50% reduction should be considered a “substantial improvement.” These thresholds, primarily derived from patient-reported outcomes on the NRS and VAS, have become widely used in FDA pain trials [14]. IMMPACT further emphasized the importance of utilizing responder analyses, which categorize participants as “responders” or “non-responders” based on whether they meet specific thresholds. For example, instead of stating that a treatment caused an average reduction of 1.2 points, researchers can say that “40% of patients achieved at least a 30% reduction in pain” [29]. This framing makes trial outcomes more tangible for both patients and clinicians and directly applicable to clinical decision-making.

Despite their advantages, responder thresholds pose several methodological and interpretive challenges. They rely on arbitrary cutoffs that may not fully represent the spectrum of meaningful improvement. For example, a patient with a 29% reduction is categorized as a “non-responder,” while someone with a 31% reduction is a “responder,” even if their experiences are clinically similar. This “all-or-none” dichotomy can overlook correlations that may have a continuous impact on outcomes [29]. Responder analyses can be statistically inefficient, resulting in reduced trial power compared to continuous measures. Converting continuous data into binary categories neglects any variability that could otherwise strengthen inference [29]. This increases the risk of underestimating treatment effects or failing to detect important subgroup differences. Additionally, responder definitions may not be applicable across different pain conditions. What constitutes meaningful improvement in chronic neuropathic pain may differ from that in acute musculoskeletal pain. The variety of pain mechanisms suggests thresholds like ≥30% or ≥50% could be too strict if applied universally [29]. As mentioned, responder thresholds may not perfectly match patients’ priorities. Some individuals value even small reductions in pain if accompanied by improvements in sleep or function, while others expect almost complete relief before considering a treatment worthwhile. This complexity indicates the need to pair responder definitions with patient global impression scales, functional outcomes, or composite endpoints that more accurately reflect the multidimensional nature of pain [28,30].

## 6. Bridging the Efficacy–Effectiveness Gap

As previously mentioned, the efficacy–effectiveness gap reflects differences in patient populations, treatment adherence, concomitant interventions, and outcome measurement between trials and clinical practice [10,11]. The methodological rigor that maximizes internal validity in trials is rarely replicated in everyday practice, creating significant uncertainty when applying trial results to individual patients [10,18].

The problem is magnified in chronic pain, where Koechlin et al. (2024) demonstrated apparent discrepancies between synthesized trial evidence and real-world prescribing for chronic primary musculoskeletal pain [15]. At the same time, high-quality real-world studies on neuropathic pain suggest that when outcomes and populations are carefully defined, RWD can complement trial evidence and provide clinically relevant implications [5]. Therefore, the task for pain medicine is not to replicate trials in practice, but rather to integrate the trial elements essential for generating reliable and interpretable evidence into routine care [31].

Pain medicine has increasingly recognized that pain intensity is a necessary but insufficient endpoint. The burden of chronic pain affects function, sleep, mood, and quality of life, and is best revealed through PROs [11,31]. PROs, including the BPI, ODI, and Short Form-36 (SF-36), offer validated insights into how patients perceive treatment benefits [24]. In trials, incorporating PROs has shifted the field toward a multidimensional, patient-centric assessment of efficacy [17]. This same shift is needed in routine practice. Systematic PRO collection is beneficial because it facilitates shared decision-making, helping clinicians interpret changes not only in pain levels but also regarding whether patients achieve outcomes they consider meaningful (e.g., restored mobility, better sleep) [28]. Additionally, it enhances the quality of real-world data by ensuring that analyses reflect domains that are important to patients, rather than relying on surrogate measures [11,17,31]. When framed in responder terms, such as a ≥50% reduction in pain and improvement on the Patient Global Impression of Change (PGIC) scale, PROs provide a clear language that patients, clinicians, and regulators can all interpret consistently. Routinely including PROs in clinical care can align research priorities with patients’ lived experiences and strengthen the external validity of trial results.

One of the most direct strategies for narrowing the efficacy–effectiveness gap is to implement structured, validated outcome measurements in routine clinical practice. Incorporating these outcomes within the EMR as electronic PROs and collecting them at standardized intervals (i.e., baseline, 3, 6, and 12 months) allows clinicians to interpret progress in responder terms (i.e., ≥30% or ≥50% improvement). This approach is both intuitive for patients and consistent with regulatory guidelines of clinically meaningful benefit [10,18]. Utilizing a systematic approach to collect this RWD can generate datasets that resemble quality evidence found in RCTs and improve clinical decision-making across health systems. At a minimum, outcome measurement should include pain intensity, pain interference or disability, and overall impressions of change, as outlined in Table 3.

The placebo effect was previously viewed as a confounder in RCTs but is increasingly recognized as a clinically meaningful and validated component of the overall treatment effect [31,32]. In pain medicine, factors such as outcome expectation, conditioning, patient–clinician rapport, and therapeutic continuity all influence the strength of placebo responses [32,33]. Instead of dismissing these mechanisms, clinicians can intentionally and ethically incorporate them into routine care. Strategies include framing treatment communication positively and emphasizing the potential for improvement, enhancing therapeutic processes through consistent pre-treatment routines and structured follow-up, and maintaining empathy and clinician engagement throughout all patient interactions. Evidence suggests that these approaches can augment patient-reported improvement and reinforce compliance, which enhances real-world treatment outcomes closer to those observed in controlled trials [31,32,33]. Documenting expectations and PGIC responses alongside symptom scores may help providers differentiate between pharmacological effects and contextual ones. In doing so, clinicians would be able to strengthen both the treatment itself and the patient–clinician relationship [31,33].

## 7. Implications for the Pain Practice

### 7.1. Role of Combined RCT and RWD Evidence in Policy, Reimbursement, and Guidelines

The integration of RCT and RWD evidence creates a complementary framework that provides more effective information for health policy, reimbursement decisions, and the development of clinical guidelines. Evidence syntheses in pain medicine that incorporate both trial and real-world findings allow guideline committees to make recommendations that balance internal and external validity [1,30]. From a reimbursement perspective, payers are shifting toward value-based models that reward treatments demonstrating durable, patient-centered benefits across broad populations. In this context, RWD offers the necessary background for assessing cost-effectiveness and equity, while RCTs provide the foundational proof of efficacy. Together, these data sources influence coverage decisions that are both scientifically valid and economically sustainable [5,15]. Regulatory agencies, such as the FDA, are increasingly incorporating RWE in post-marketing safety surveillance and label expansions, further integrating the hybrid evidence approach into the policy landscape [1,3,19].

### 7.2. Potential for Improving Equity in Pain Treatment Outcomes

Equity in pain management has long been compromised by the underrepresentation of racial and ethnic minority groups, individuals from lower socioeconomic backgrounds, and those with multiple health conditions in RCTs. This lack of representation has contributed to inequitable treatment recommendations and unequal outcomes across these populations. By evaluating results from broader and more heterogeneous patient groups, RWD becomes a valuable tool for identifying disparities in treatment effectiveness and access, adherence patterns, side effect profiles, and functional outcomes [3,11,15]. For example, real-world analyses may reveal that specific pharmacologic interventions are less tolerated in patients with high comorbidity burdens or that adherence challenges are more apparent in socially disadvantaged groups [34]. These not only reveal gaps in current care delivery but also guide targeted systemic interventions aimed at improving access and outcomes.

Integrating PROs further strengthens equity-focused research. PROs ensure that outcome measurements reflect what patients themselves value most, which may differ across cultural, social, and demographic contexts [17]. For example, while some groups may prioritize pain relief above all else, others may consider functional restoration, work participation, or sleep improvement as the most important benefits. Incorporating PROs within both trial design and RWE methodology allows for a more comprehensive and inclusive assessment of treatment success. By incorporating equity-focused analyses into both RCT interpretation and RWD research, the field of pain medicine can progress toward treatment strategies that are not only evidence-based but also more patient-centric and equitable. These approaches ensure that clinical guidelines, reimbursement frameworks, and policies acknowledge diverse patient experiences and do not perpetuate care inequities.

## 8. Conclusions

Chronic pain research benefits from both randomized controlled trials and real-world data. While RCTs remain essential for demonstrating causal efficacy, their narrow design often limits applicability to everyday patient populations. In contrast, RWD offers a better understanding of long-term outcomes, safety concerns, and variations in treatment response across heterogeneous groups. Utilizing both approaches can strengthen the evidence base and ensure that therapies are rooted in methodological rigor while also reflecting clinical diversity. Incorporating validated patient-reported outcomes and clinically relevant benchmarks further aligns research with patient priorities and enhances interpretability. A more deliberate integration of these data sources will support informed policy decisions, equitable guideline development, and personalized pain management. Ultimately, combining trial precision with the inclusivity of real-world evidence can lead to more reliable and patient-centered solutions in chronic pain.

## Figures and Tables

**Table 1:** Comparison of randomized control trials and real-world data across several domains.

	Randomized Control Trials (RCTs)	Real-World Data (RWD)
Population	Narrow, homogeneous; often excludes comorbidities and polypharmacy	Broad, heterogeneous; reflects real-world complexity
Validity Focus	High internal validity; causal inference under controlled conditions	High external validity; effectiveness in routine care
Primary Outcomes	Short-term efficacy; standardized endpoints (i.e., Numeric Rating Scale [NRS]/Visual Analogue Scale [VAS])	Multidimensional outcomes including function and Patient-Reported Outcomes [PROs]
Long-Term Safety Analysis	Often underpowered for rare or delayed events	Rare and delayed events can be detectable in large cohorts
Treatment Adherence	Optimized and reinforced routinely under protocol	Variable; reflects real-world patterns and patient preferences
Follow-up	Weeks to months in many trials	Months to years; suitable for durability assessments
Limitations	Limited generalizability; artificial conditions	Confounding by indication; variable data quality

Numeric Rating Scale [NRS]; Visual Analogue Scale [VAS]; Patient-Reported Outcomes [PROs].

**Table 2:** Sources of the efficacy–effectiveness gap from both RCTs and RWD [11,18].

Efficacy-Effectiveness Gap
Patient Population - Exclusion of older adults, multimorbidity, psychiatric comorbidities, polypharmacy, socioeconomic complexity
Trial Design & Setting - Structured treatment adherence and monitoring - Standardized follow-up protocols - Limited scope and short duration - Placebo effect
Measurement Limitations - Reliance on unidimensional pain scales - Underrepresentation of functional, psychosocial, and quality-of-life outcomes - Lack of ecological validity
Healthcare System & Practice - Differences in clinician expertise and preferred dosing strategies - Inconsistent access to multidisciplinary care - Resource constraints and systemic barriers
Sociocultural & Behavioral Factors - Variability in treatment adherence - Influence of psychosocial context, patient expectations, and cultural differences

**Table 3:** Minimum outcome measurements to bridge the efficiency–effectiveness gap.

Outcome Measurement	Common Instruments	Description
Pain Intensity	NRS, VAS	Evaluates the severity of pain on a unidimensional scale; simple, validated, and sensitive to clinical change
Pain Interference/Disability	BPI, ODI, RMDQ	Assesses the extent to which pain disrupts daily function, mobility, and quality of life
Impressions of Change	PGIC, MCID	Provides an overall, patient-centric measure of perceived benefit and clinical meaningfulness

Note: NRS = Numerical Rating Scale; VAS = Visual Analog Scale; BPI = Brief Pain Inventory; ODI = Oswestry Disability Index; RMDQ = Roland Morris Disability Questionnaire; PGIC = Patient Global Impression of Change; MCID = Minimal Clinically Important Difference.

## Data Availability

No new data were created or analyzed in this study. Data sharing is not applicable to this article.

## Abbreviations

The following abbreviations are used in this manuscript:

RCT	Randomized Control Trial
RWD	Real-World Data
RWE	Real-World Evidence
FDA	U.S. Food and Drug Administration
EMR	electronic medical record
PRO	patient reported outcome
PSM	Propensity score matching
VAS	Visual Analog Scale
NRS	Numerical Rating Scale
PRPPR	Patient-reported Percentage Pain Reduction
CPPR	Calculated Percentage Pain Reduction
MPQ	McGill Pain Questionnaire
BPI	Brief Pain Inventory
ODI	Oswestry Disability Index
RMDQ	Roland–Morris Disability Questionnaire
MCID	Minimal Clinically Important Difference
IMMPACT	Initiative on Methods, Measurement, and Pain Assessment in Clinical Trials
SF-36	Short Form-36
PGIC	Patient Global Impression of Change
